# The Performance of Deep Neural Networks in Differentiating Chest X-Rays of COVID-19 Patients From Other Bacterial and Viral Pneumonias

**DOI:** 10.3389/fmed.2020.00550

**Published:** 2020-08-18

**Authors:** Mohamed Elgendi, Muhammad Umer Nasir, Qunfeng Tang, Richard Ribon Fletcher, Newton Howard, Carlo Menon, Rabab Ward, William Parker, Savvas Nicolaou

**Affiliations:** ^1^School of Electrical and Computer Engineering, University of British Columbia, Vancouver, BC, Canada; ^2^Department of Obstetrics & Gynaecology, Faculty of Medicine, University of British Columbia, Vancouver, BC, Canada; ^3^BC Children's & Women's Hospital, Vancouver, BC, Canada; ^4^School of Mechatronic Systems Engineering, Simon Fraser University, Burnaby, BC, Canada; ^5^Nuffield Department of Surgical Sciences, University of Oxford, Oxford, United Kingdom; ^6^Department of Emergency and Trauma Radiology, Vancouver General Hospital, Vancouver, BC, Canada; ^7^D-Lab, Massachusetts Institute of Technology, Cambridge, MA, United States; ^8^Department of Radiology, Faculty of Medicine, University of British Columbia, Vancouver, BC, Canada

**Keywords:** chest X-ray radiography, artificial intelligence, image classification, neural network, convolutional neural networks, corona virus, transfer learning

## Abstract

Chest radiography is a critical tool in the early detection, management planning, and follow-up evaluation of COVID-19 pneumonia; however, in smaller clinics around the world, there is a shortage of radiologists to analyze large number of examinations especially performed during a pandemic. Limited availability of high-resolution computed tomography and real-time polymerase chain reaction in developing countries and regions of high patient turnover also emphasizes the importance of chest radiography as both a screening and diagnostic tool. In this paper, we compare the performance of 17 available deep learning algorithms to help identify imaging features of COVID19 pneumonia. We utilize an existing diagnostic technology (chest radiography) and preexisting neural networks (DarkNet-19) to detect imaging features of COVID-19 pneumonia. Our approach eliminates the extra time and resources needed to develop new technology and associated algorithms, thus aiding the front-line healthcare workers in the race against the COVID-19 pandemic. Our results show that DarkNet-19 is the optimal pre-trained neural network for the detection of radiographic features of COVID-19 pneumonia, scoring an overall accuracy of 94.28% over 5,854 X-ray images. We also present a custom visualization of the results that can be used to highlight important visual biomarkers of the disease and disease progression.

## 1. Introduction

On March 11, 2020, the World Health Organization declared the COVID-19 virus as an international pandemic (1). The virus spreads among people via physical contact and respiratory droplets produced by coughing or sneezing (2). The current gold standard for diagnosis of COVID-19 pneumonia is real-time reverse transcription-polymerase chain reaction (RT-PCR). The test itself takes about 4 h; however, the process before and after running the test, such as transporting the sample and sending the results, requires a significant amount of time. Pertaining to PCR testing is not a panacea, as the sensitivities range from 70 to 98% depending on when the test is performed during the course of the disease and the quality of the sample. In certain regions of the world it is simply not routinely available. More importantly, the RT-PCR average turnaround time is 3–6 days, and it is also relatively costly at an average of CA*$*4, 000 per test (3). The need for a faster and relatively inexpensive technology for detecting COVID-19 is thus crucial to expedite universal testing.

The clinical presentation of COVID-19 pneumonia is very diverse, ranging from mild to critical disease manifestations. Early detection becomes pivotal in managing the disease and limiting its spread. In 20% of the affected patient population, the infection may lead to severe hypoxia, organ failure, and death (4). In order to meet this need, high-resolution computed tomography (HRCT) and chest radiography (CR, known as chest X-ray imaging) are commonly available worldwide. Patterns of pulmonary parenchymal involvement in COVID-19 infection and it's progression in the lungs has been described in multiple studies (5). However, despite the widespread availability of X-ray imaging, there is unfortunately a shortage of radiologists in most low-resource clinics and developing countries to analyze and interpret these images. For this reason, artificial intelligence and computerized deep learning that can automate the process of image analysis have begun to attract great interest (6). Note that X-ray costs about CA*$*40 per test (3), making it a cost effective and readily available option. Moreover, the X-ray machine is portable, making it versatile to be utilized in all areas of the hospital even in the Intensive Care Unit.

Since the initial outbreak of the COVID-19, a few attempts have been made to apply deep learning to radiological manifestations of COVID-19 pneumonia. Narin et al. (7) reported an accuracy of 98% on a balanced dataset for detecting COVID-19 after investigating three pre-trained neural networks. Sethy and Behera (8) explored 10 different pre-trained neural networks, reporting an accuracy of 93% on a balanced dataset, for detecting COVID-19 on X-ray images. Zhang et al. (9) utilized only one pre-trained neural network, scoring 93% on an unbalanced dataset. Hemdan et al. (10) looked into seven pre-trained networks, reporting an accuracy of 90% on a balanced dataset. Apostolopoulos and Bessiana (11) evaluated five pre-trained neural networks, scoring 98% of accuracy on an unbalanced dataset.

However, these attempts did not make clear which existing deep learning method would be the most efficient and robust for COVID-19 compared to many others. Moreover, some of these studies were carried out on unbalanced datasets. Note that an unbalanced dataset is a dataset where the number of subjects in each class is equal. Our study aims to determine the optimal learning method, by investigating different types of pre-trained networks on a balanced dataset, for COVID-19 testing. Additionally, we attempt to visualize the optimal network weights, which were used for decision making, on top of the original X-ray image to visually represent the output of the network.

## 2. Method

We investigated 17 pre-trained neural networks: AlexNet, SqueezNet (12), GoogleNet (13), ResNet-50 (14), DarkNet-53 (15), DarkNet-19 (15), ShuffleNet (16), NasNet-Mobile (17), Xception (18), Place365-GoogLeNet (13), MobileNet-v2 (19), DenseNet-201 (20), ResNet-18 (14), Inception-ResNet-v28 (21), Inception-v3 (22), ResNet-101 (14), and VGG-19 (23).

All the experiments in our work were carried out in MATLAB 2020a on a workstation (GPU NVIDIA GeForce RTX 2080Ti 11 GB, RAM 64 GB, and Intel Processor I9-9900K @3.6 GHz). The dataset was divided into 80% training and 20% validation.

The last fully connected layer was changed into the new task to classify two classes. The following parameters were fixed for the 17 pre-trained neural networks: learning rate was set to 0.0001, validation frequency was set to 5, max epochs was set to 8, and the min batch size was set to 64.

The class activation mapping was carried by multiplying the image activations from the last ReLU layer by the weights of the last fully connected layer of the DarkNet-19 network, called “leaky18,” as follows:

(1) $$\begin{array}{l} C\left(x,y\right)=\sum W_{l=61}F_{l=60}\left(x,y\right) \end{array}$$

where *C* is the class activation map, *l* is the layer number, *F* is the image activations from ReLu layer (*l* = 60) with dimensions of 8 × 8 × 1, 024. Here, *W* refers to the weights at *l* = 61 with dimensions of 1 × 1 × 1, 024. Thus, the dimensions of *C* is 8 × 8. We then resized *C* to match the size of the original image and visualized it using a jet colormap.

## 3. Dataset Used

Two datasets are used, the first dataset is the publicly available CoronaHack-Chest X-Ray-Dataset which can be downloaded from this link: https://www.kaggle.com/praveengovi/coronahack-chest-xraydataset. This dataset contains the following number of images: 85 COVID-19, 2,772 bacterial, and 1,493 viral pneumonias. The second dataset is a local dataset collected from an accredited Level I trauma center: Vancouver General Hospital (VGH), British Columbia, Canada. The dataset contains only 85 COVID X-ray images.

### 3.1. Dataset 1: Training and Validation

The CoronaHack -Chest X-Ray-Dataset contains only X-ray 85 images for COVID, and to balance the dataset for neural network training, we had to downsize the sample size from 85 to 50 by random selection. To generate the “other class,” we downsized the samples, by selecting 50 radiographic images that were diagnosed as healthy to match and balance the COVID-19 class. Radiographs labeled as bacterial or other viral pneumonias have also been included in the study to assess specificity. The number of images used in training and validation to retrain the deep neural network is shown in Table 1.

**Table 1:** Number of X-ray images used for training, validation, and testing in this study.

**Dataset**			**Class 1**	**Class 2**		
**Name**	**Purpose**	**Location of origin**	**COVID**	**Healthy**	**Pneumonia bacterial**	**Pneumonia viral**
Dataset 1	Training	CoronaHack -Chest X-Ray-Dataset	40	13	13	14
Dataset 1	Validation	CoronaHack -Chest X-Ray-Dataset	10	3	3	4
Dataset 2	Testing	CoronaHack -Chest X-Ray-Dataset and Vancouver General Hospital	58	1,560	2,761	1,475
Total number of X-ray images			108	1,576	2,777	1,493

### 3.2. Dataset 2: Testing

Data collected from the Vancouver General Hospital (VGH) contained 58 chest radiographs with pulmonary findings ranging from subtle to severe radiographic abnormality, which was confirmed by two radiologists individually on visual assessment with final interpretations with over 30 years of radiology experience combined. These 58 radiographs were obtained from 18 RT-PCR-positive COVID-19 patients. Serial radiographs acquired during a patient's hospital stay showing progressive disease were also included in the data set. The data set contained anteroposterior and posteroanterior projections. Portable radiographs acquired in intensive care units with lined and tubes in place were also included in the data set. The images (true positive) submitted for the analysis by the VGH team were anonymized and mixed with an equal number of normal chest radiographs to create a balanced data set.

The remaining from the CoronaHack-Chest X-Ray-Dataset was used to test the specificity of the algorithm. Dataset 2 was used an external dataset to test the robustness of the algorithm, with a total of 5,854 X-ray images (58 COVID-19, 1,560 healthy, 2,761 bacterial, and 1,475 viral pneumonias), as shown in Table 1. Note that there is no overlap between Dataset 1 and Dataset 2.

## 4. Results and Discussion

**Figure 1:**
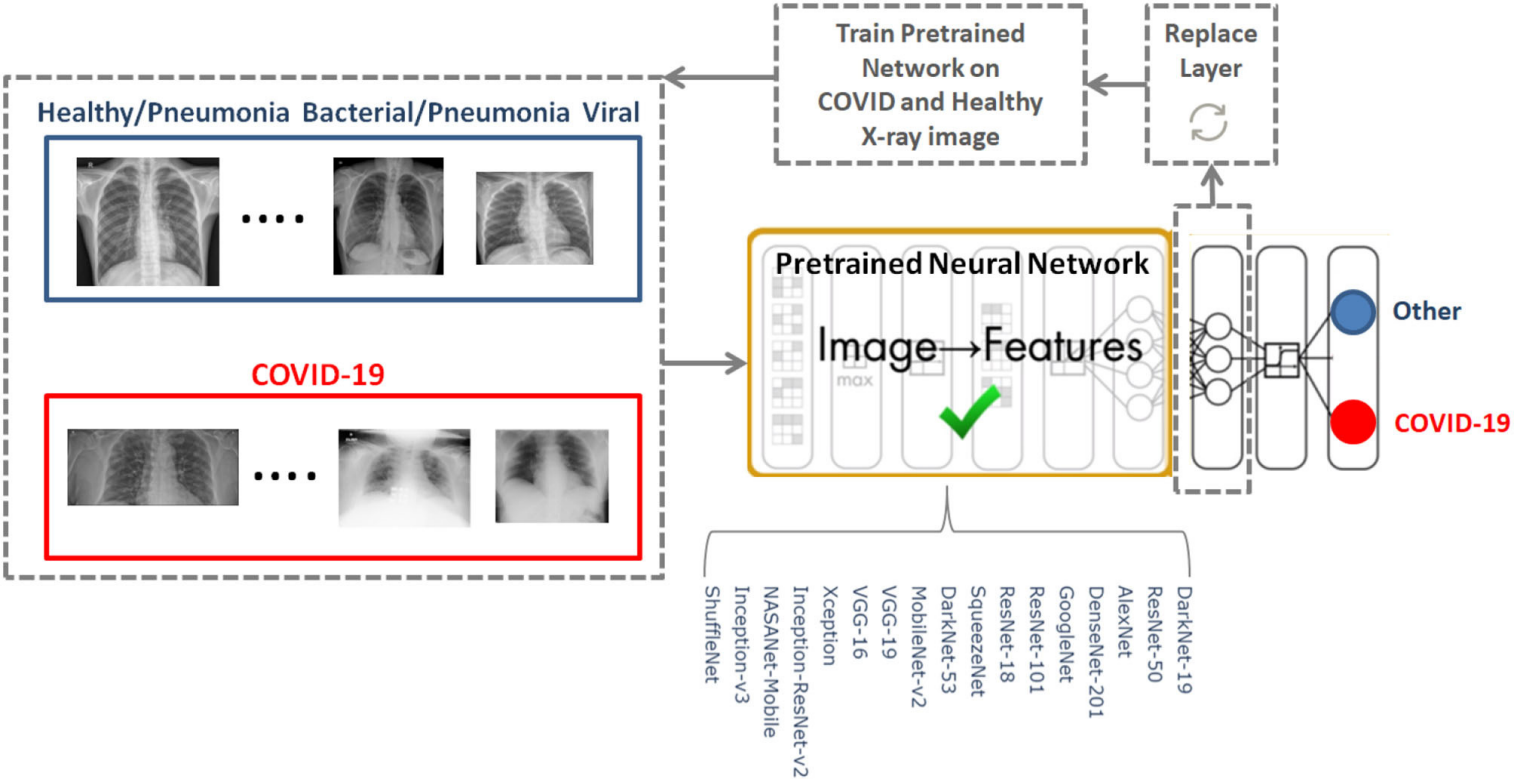
COVID-19 detection framework using pre-trained neural networks.

To determine the optimal existing pre-trained neural network for the detection of COVID-19, we used the CoronaHack-Chest X-Ray-Dataset. The chest X-ray images dataset contains 85 images from patients diagnosed with COVID-19 and 1,576 images from healthy subjects. Five X-ray images collected from the Lateral position were deleted for consistency. We then balanced the dataset to include 50 RT-PCR positive COVID-19 patients and 50 healthy subjects. From the group of 85 RT-PCR positive cases patients were randomly selected with varying extent of pulmonary parenchymal involvement. After creating a balanced dataset, which is important for producing solid findings, 17 pre-trained networks were analyzed following the framework shown in Figure 1.

The 17 pre-trained neural networks were trained on a large data set by using more than a million images, as a result the algorithms developed can classify new images into 1,000 different object categories, such as keyboard, mouse, pencil, and various animals. Through artificial intelligence and machine learning each network can detect images based on unique features representative of a particular category. By replacing the last fully connect layer, as shown in Figure 1, and retraining (fine-tune deeper layers) the neural network with the new dataset (50 COVID-19 and 50 other), the neural network can detect COVID-19 and other populations.

The performance of 17 pre-trained neural networks using the same dataset (50 COVID-19 and 50 other), is shown in Table 2. Interestingly, we found that the following two pre-trained neural networks achieved an accuracy of 100% during the training and validation phases using Dataset 1: ResNet-50 and DarkNet-19.

**Table 2:** Performance of 17 pre-trained neural networks on Dataset 1.

**Network**	**Training accuracy**	**Validation accuracy**	**Overall performance**
DarkNet-19	100.00	100.00	100.00
ResNet-50	100.00	100.00	100.00
AlexNet	100.00	95.00	97.50
DenseNet-201	100.00	95.00	97.50
GoogleNet	100.00	95.00	97.50
ResNet-101	100.00	95.00	97.50
ResNet-18	100.00	95.00	97.50
SqueezeNet	100.00	95.00	97.50
DarkNet-53	100.00	90.00	95.00
MobileNet-v2	100.00	90.00	95.00
VGG-19	100.00	90.00	95.00
VGG-16	90.91	95.00	92.95
Xception	90.91	95.00	92.95
Inception-ResNet-v2	81.82	100.00	90.91
NASANet-Mobile	81.82	100.00	90.91
Inception-v3	100.00	80.00	90.00
ShuffleNet	90.91	85.00	87.95

*The neural networks are ranked in descending order based on the overall performance on the training and validation images*.

Inception-v3 and ShuffleNet achieved an overall validation accuracy below 90% suggesting that these neural networks are not robust enough for detecting COVID-19 compared to, for example, ResNet-50 and DarkNet-19. Despite that the Inception-ReNet-v2 was pre-trained on trained on more than a million images from the ImageNet database (21), it was not ranked the highest in terms of the overall performance, suggesting it is not suitable to use for detecting COVID-19.

**Figure 2:**
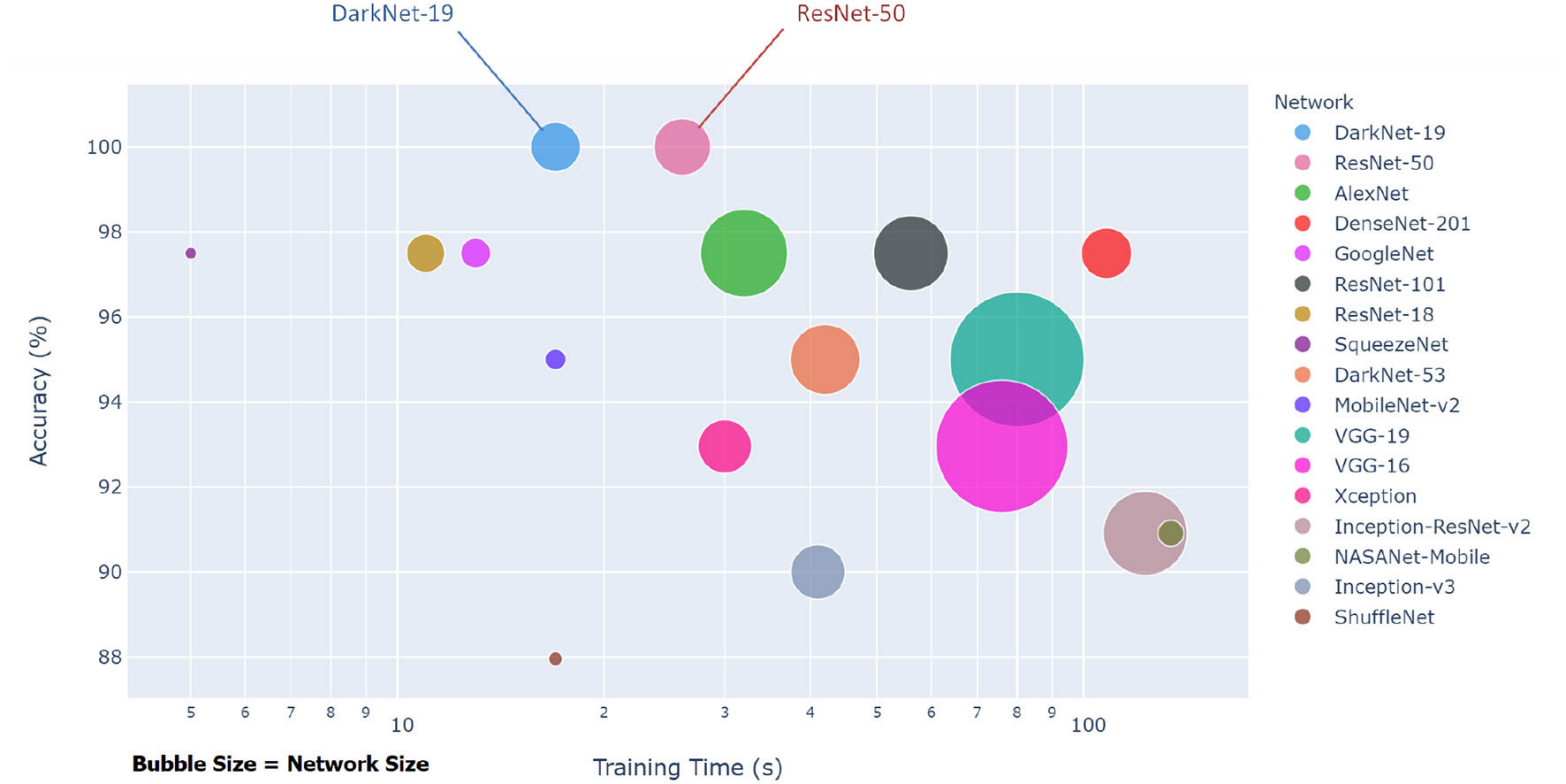
Overall performance of 17 pre-trained neural networks for detecting COVID-19 using Dataset 1.

Each pre-trained network has a structure that is different from others, e.g., number of layers and size of input. The most important characteristics of a pre-trained neural network are as follows: accuracy, speed, and size (24). Greater accuracy increases the specificity and sensitivity for COVID-19 detection. Increased speed allows for faster processing. Smaller networks can be deployed on systems with less computational resources. Therefore, the optimal network is the network that increases accuracy, utilizes less training time, and that is relatively small. Typically, there is a tradeoff between the three characteristics, and not all can be satisfied at once. However, our results show that it is possible to satisfy all three requirements. DarkNet-19 outperformed all other networks, while having increased speed and increased accuracy in a relatively small-sized network, as shown in Figure 2. A visual comparison between all investigated pre-trained neural networks is presented, with respect to the three characteristics. The x-axis is the training time (logarithmic scale) in seconds, the y-axis is the overall validation accuracy and the bubble size represents the network size. Note that DarkNet-19 and ResNet-50 achieved an accuracy of 100%; however, DarkNet is much faster and requires less memory.

A comparison of optimal neural networks recommended in previous studies, along with the optimal neural network suggested by this work, is shown in Table 3. Narin et al. (7) used a balanced sample size of 100 subjects (50 COVID-19 and 50 healthy). They investigated three pre-trained neural networks: ResNet50, InceptionV3 and InceptionResNetV2, with a cross validation ratio of 80–20%. They found that ResNet50 outperformed the other two networks, scoring a validation accuracy of 98%.

**Table 3:** Comparison between optimal pre-trained neural networks proposed for detecting COVID-19 to date.

**Study**	**Optimal network**	**COVID-19 sample size**	**Other sample size**	**Cross validation (%)**	**Validation accuracy (%)**
This work	DarkNet-19 and ResNet-50	50	50	80–20	100
Narin et al. (7)	ResNet-50	50	50	80–20	98
Sethy and Behera (8)	ResNet-50 + SVM	25	25	80–20	95
Zhang et al. (9)	ResNet-50	70	30	NC	96
Hemdan et al. (10)	VGG19 and DenseNet201	25	25	80–20	90
Apostolopoulos and Bessiana (11)	VGG-19	224	504	90–10	98.75

*SVM refers to Support Vector Machine while NC refers to not clear*.

Sethy and Behera (8) used a balanced sample size of 50 subjects (25 COVID-19 and 25 healthy). They extracted features from pre-trained neural networks and fed them to Support vector Machine (SVM) for classification. They explored the following pre-trained neural networks: AlexNet, DenseNet201, GoogleNet, Inceptionv3, ResNet18, ResNet50, ResNet101, VGG16, VGG19, XceptionNet, and Inceptionresnetv2, with a cross validation ratio of 80–20%. Again, ResNet50 in combination with SVM outperformed the other networks, with a validation accuracy of 95%.

A similar study by Hemdan et al. (10) used a balanced sample size of 50 subjects (25 COVID-19 and 25 healthy). The following pre-trained neural networks were evaluated: VGG19, DenseNet201, InceptionV3, ResNetV2, InceptionResNetV2, Xception, and MobileNetV2, with a cross validation ratio of 80–20%. Both VGG19 and DenseNet201 scored the same validation accuracy of 90%.

Two studies reported results based on unbalanced datasets: Zhang et al. (9) and Apostolopoulos and Bessiana (11). Zhang et al. (9) created a deep learning network based on ResNet-50, which achieved an accuracy of 96% with a dataset of 70 COVID-19 and 30 Healthy subjects. Apostolopoulos and Bessiana (11) used a sample size of 224 COVID-19 and 504 healthy subjects. They tested five pre-trained neural networks: VGG19, InceptionV3, InceptionResNetV2, Xception, and MobileNetV2. They found that VGG19 scored highest accuracy of 98.75%, with a cross validation ratio of 90–10%.

It is worth noting that the studies discussed in Table 3 did not use other populations, such as pneumonia bacterial to test specificity. Moreover, they did not use an external dataset to test reliability. In other words, they had only training and validation datasets. Note that we used two datasets: Dataset 1 for training and validation and Dataset 2 for testing. Interestingly, ResNet-50 network achieved a high accuracy in three different studies. Note that these studies only compared ResNet-50 to a select few neural networks, whereas here we compared a total of 17. One possible reason that our ResNet-50 achieved 100% is that the dataset (Dataset 1) in our study differed from the datasets in other studies. Another reason is the network's parameter settings (e.g., learning rate). However, DarkNet-19 also achieved a validation accuracy of 100%, and it is not clear which network is more accurately detect radiographic abnormalities associated with COVID-19 pneumonia.

## 5. DarkNet-19 Vs. ResNet-50

Two approaches will be used to compare the performance between the DarkNet-19 and ResNet-50 networks: (1) model fitting and (2) performance over Dataset 2.

**Figure 3:**
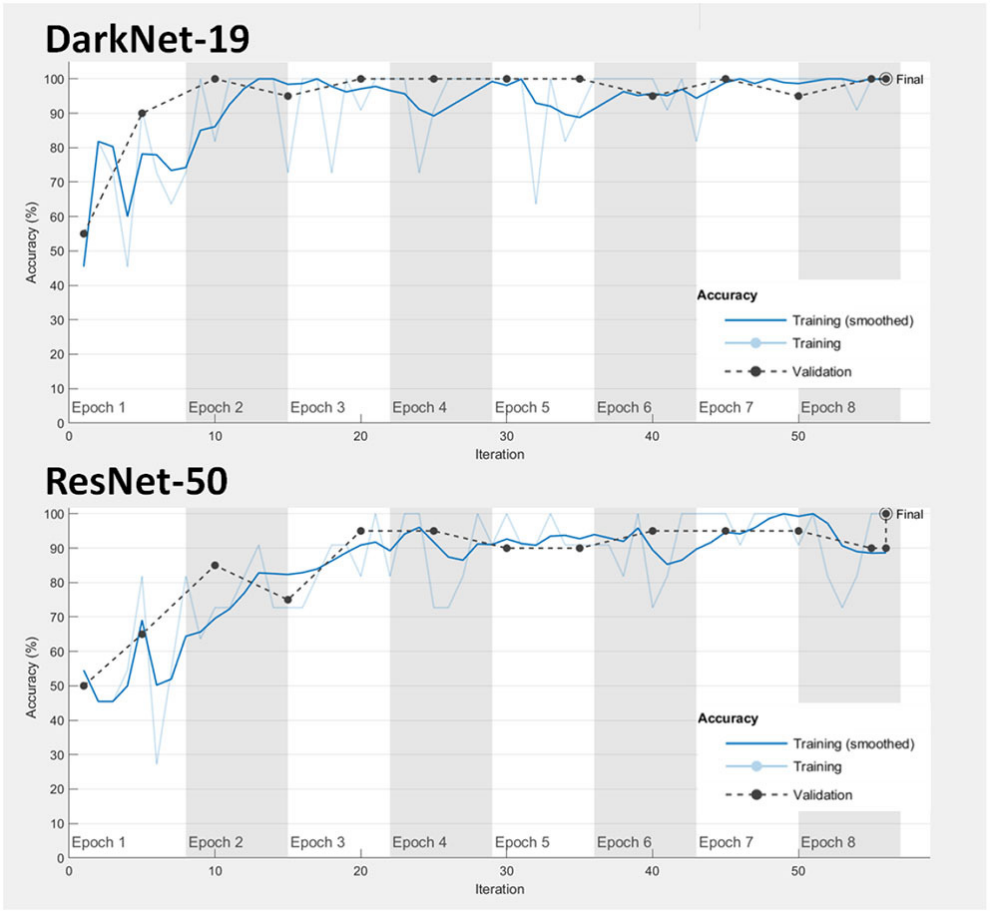
Learning performance of DarkNet-19 and ResNet-50.

1. **Model fitting:** Achieving a good model fitting is the target behind any learning algorithm by providing a model that does not suffer from either over-fitting and under-fitting (25). Typically, a “good fitted” model is obtained when both training and validation loss curves decrease to a stability zone where the gap between the loss curves is minimal (25). This gap is referred to as the “generalization gap,” and it can be seen in Figure 3; the gap between the loss curves in DarkNet-19 is smaller than the gap in ResNet-50. This suggests that DarkNet-19 is more optimal when compared to ResNet-19 even though both achieved 100% accuracy of the training and validation images using Dataset 1.
2. **Performance over the testing dataset:** In this step, the reliability and robustness of DarkNet-19 and ResNet-50 over Dataset 2 will be examined. As can be seen in Table 4, both neural networks were able to differentiable the pattern of COVID-19 pneumonia from other patterns labeled as bacterial and other viral pneumonia with an accuracy >90%. However, DarkNet-19 achieved an accuracy of 96.55% while ResNet-50 achieved an accuracy of 86.21% in detecting COVID-19. In other words, DarkNet-19 outperformed ResNet-50 in terms of sensitivity. On the other hand, ResNet-50 slightly outperformed DarkNet-19 in terms of specificity.

**Table 4:** Performance comparison between DarkNet-19 and ResNet-50 over the testing dataset (Dataset 2).

**Testing dataset**	**DarkNet-19**	**ResNet-50**
**Dataset 2**	**Accuracy (%)**	**Accuracy (%)**
COVID (*n* = 58)	96.55	86.21
Other (Healthy, *n* = 1, 560)	94.53	95.29
Other (Pneumonia bacterial, *n* = 2, 756)	92.60	97.82
Other (Pneumonia viral, *n* = 1, 475)	93.44	95.44
Overall	94.28	93.69

As we are interested in finding the model that achieved high sensitivity with minimal generalization gap, the optimal neural network to be used is the DarkNet-19.

## 6. Clinical Perspective

Availability of efficient algorithms to detect and categorize abnormalities on chest radiographs into subsets can be a useful adjunct in the clinical practice. Darknet-19's accuracy to detect radiographic patterns associated with COVID-19 in portable and routine chest radiographs at varied clinical stages makes it a robust and useful tool. Use of such efficient algorithms in everyday clinical practice can help address the problem of shortage of skilled manpower, contributing to provision of better clinical care. More institution-based research is, however, required in this area.

While the DarkNet-19 algorithm can distinguish COVID-19 patients from other populations with 94.28% accuracy, we note the following limitations:

1. The COVID sample size used in the training and validation phase was relatively small, 50 images.
2. The images were not segregated based on the technique of acquisition (portable or standard supine AP chest radiograph) or positioning (posteroanterior vs. anteroposterior). Thus, any possible errors that might arise because of the patient's positioning have not been addressed in the study. Lateral chest radiographs were excluded from the data set.
3. Our investigation compared radiographic features of COVID-19 patients to healthy individuals. As a next step in our investigation, the radiographic data from COVID-19 patients should also be compared with other respiratory infections in order to improve the specificity of the algorithm for detection of COVID-19.

**Figure 4:**
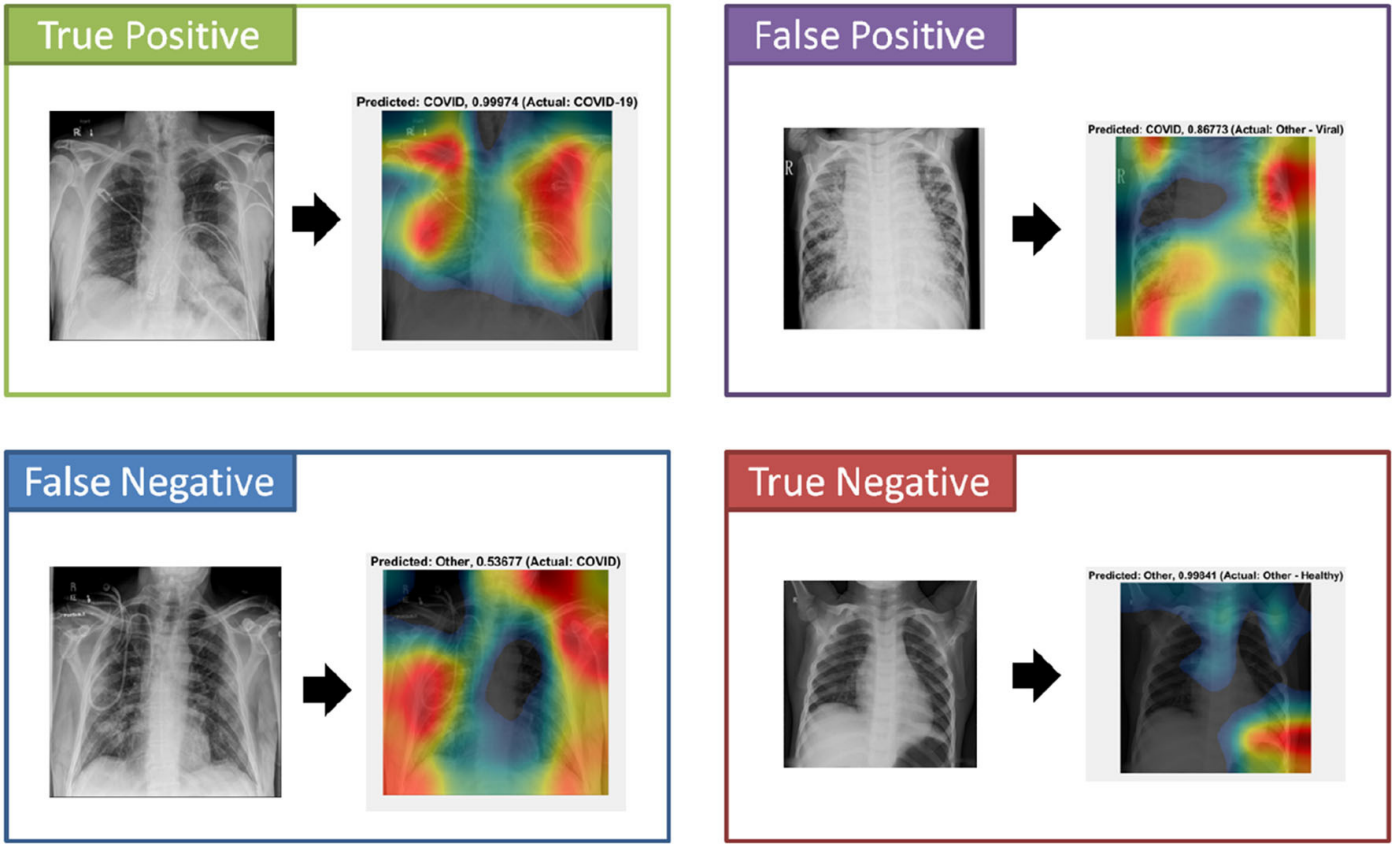
Performance examples of visualization diagnosis using class activation mapping.

An important component to the automated analysis of the X-ray data is the visualization of the X-ray images, using colors to identify the critical visual biomarkers as well as indication of disease progression. This step can make disease identification more intuitive and easier to understand, especially for healthcare workers with minimal knowledge about COVID-19. The visualization can also expedite the diagnosis process. As shown in Figure 4 (True Positive), COVID-19 subjects were identified based on the activation images and weights. Also, examples for false positive (a non-COVID subject identified as a COVID), false negative (a COVID subject identified as non-COVID), and true negative (a non-COVID subject identified as a non-COVID subject) were shown.

Note that the main purpose of this paper is not to investigate the difference between pre-trained and trained neural networks; the purpose is rather to provide a solution that is based on already existing and proven technology to use for COVID screening. If the accuracy achieved by the pre-trained neural network is not acceptable by radiologists, then exploring different untrained convolutional neural networks could be worth doing. Also, including the patient's demographic information, D-Dimer, oxygen saturation level, troponin level, neutrophil to lymphocyte ratio, glucose level, heart rate, degree of inspiration, and temperature may improve the overall detection accuracy.

## 7. Conclusion

In conclusion, fast, versatile, accurate, and accessible tools are needed to help diagnose and manage COVID-19 testing infection. The current gold standard laboratory tests are time consuming and costly, adding delays to the testing process. Chest radiography is a widely available and affordable tool for screening patients with lower respiratory symptoms or suspected COVID-19 pneumonia. Addition of computer-aided radiography can be a useful adjunct in improving throughput and early diagnosis of the disease; this is especially true during a pandemic, particularly during the surge, and in areas with a shortage of radiologists. In this paper, we have reviewed and compared many deep learning techniques currently available in the market for detecting radiographic features of COVID-19 pneumonia. After investigating 17 different pre-trained neural networks, our results showed that DarkNet-19 is the optimal pre-trained deep learning network for detection of imaging patterns of COVID-19 pneumonia on chest radiographs. Work to improve the specificity of these algorithms in the context of other respiratory infections is ongoing.

## Data Availability Statement

The CoronaHack-Chest X-Ray-Dataset used in this study is publicly available and can be downloaded from this link: https://www.kaggle.com/praveengovi/coronahack-chest-xraydataset. Requests to access the dataset collected at Vancouver General Hospital should be directed to Savvas Nicolaou, Savvas.Nicolaou@vch.ca. Dataset 1 and all trained neural networks can be accessed via this link https://github.com/Elgendi/COVID-19-Detection-Using-Chest-X-rays.

## Author Contributions

ME designed the study, analyzed the data, and led the investigation. MN, WP, and SN provided an X-ray dataset, annotated the X-ray images, and checked the clinical perspective. ME, MN, QT, RF, NH, CM, RW, WP, and SN conceived the study and drafted the manuscript. All authors approved the final manuscript.

## Conflict of Interest

The authors declare that the research was conducted in the absence of any commercial or financial relationships that could be construed as a potential conflict of interest.
